# Post-contrast T1-mapping provides a novel approach to optimal myocardial nulling for late gadolinium enhancement imaging: a quantitative prescription for the correct TI without the guesswork

**DOI:** 10.1186/1532-429X-18-S1-P22

**Published:** 2016-01-27

**Authors:** Vanessa M Ferreira, Alexander Liu, Claudia Marini, Anne Davis, Jane M Francis, Stefan Neubauer, Stefan K Piechnik

**Affiliations:** Division of Cardiovascular Medicine, Radcliffe Department of Medicine, University of Oxford, John Radcliffe Hospital, Oxford, UK

## Background

Late gadolinium enhancement (LGE) is a well-established technique that generates excellent contrast between normal and pathologic myocardium. Selecting the correct inversion time (TI) for nulling normal myocardium is essential for good quality images and accurate diagnosis. The Look-Locker (LL) TI scout is commonly used for TI estimation but is subjective and requires a long breath-hold (20 heartbeats). T1-mapping is a quantitative method that can provide a direct numeric TI prescription in a shorter breath-hold but has not been tested in practice.

## Methods

CMR: 39 subjects (19 men, 51 ± 16 yrs) with a variety of cardiac conditions underwent CMR at 1.5T (Siemens Avanto MR system; Syngo VB17A). All received intravenous gadolinium contrast (Gadoterate meglumine, Dotarem; 0.10 mmol/kg). Imaging included the LL TI scout followed immediately by post-contrast T1-mapping (ShMOLLI; WIP561; 9 heartbeats) at a mid-ventricular short-axis slice, and LGE (PSIR) imaging using conventional TI selection based on visual assessment of the TI scout according to radiographer experience.

Image processing: matching regions of interest (ROIs) were prescribed on LL TI scout, post-contrast T1-maps and LGE images in an area of the LV without obvious abnormality. LL TI was calculated by linear interpolation of sign reconstructed from the 5-6 lowest SI measurements, while T1-map TI was calculated as TI = 0.69 × T1 from post-contrast T1-maps. We also measured the average SI of the ROI on LGE images to assess for adequate nulling (SI=0) of the myocardium.

## Results

**Figure 1:**
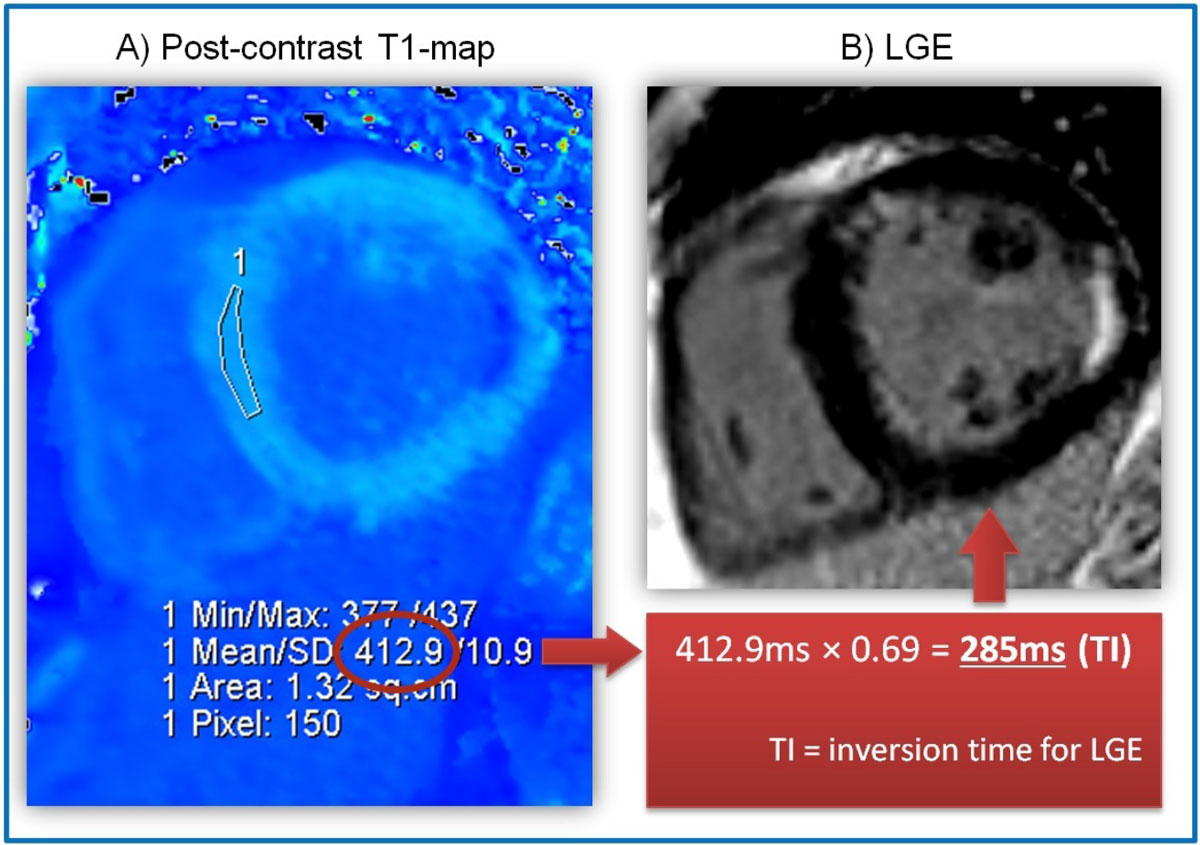
**Post-contrast T1-mapping enables prescription of a numeric TI for optimal myocardial nulling on LGE imaging directly on the CMR scanner console - illustration in a patient with prior myocardial infarction**. (A) Post-contrast T1-map, directly reconstructed on the CMR console, with a region of interest (ROI) placed in remote myocardium using standard tools in the console image viewer. The ROI provides a mean T1 value from which to calculate the inversion time: TI = 0.69 × post-contrast T1. (B) LGE image with correctly nulled myocardium acquired using TI calculated from post-contrast T1-mapping.

There was excellent correlation (r^2^=0.77, p < 0.01) between the TI calculated from LL TI scouts and post-contrast T1-maps. The LL TI was 37 ms shorter than T1map TI, consistent with the speed of T1 recovery between measurements (~15 ms/min; *Messroghli et al, MRM 2007*), combined with T1 correction for this T1-mapping sequence (*Piechnik et al, JCMR 2015*). In 18% (7/39) of the cases, the operator did not prescribe the correct TI, predominantly when the correct TI is short but the operator overestimated by assuming a starting TI of 300 ms typical for PSIR LGE imaging. Deviation of the prescribed TI from the calculated TI was correlated to suboptimal nulling of the myocardium, whether calculation of the TI was performed using ROIs on LL TI scouts (r^2^=0.34) or post-contrast T1-maps (r^2^=0.42). Figure 1 shows an example of using T1-mapping to prescribe a numerical TI, directly on the CMR console, for optimal myocardial nulling on LGE.

## Conclusions

Post-contrast T1-mapping is an easy way to provide a direct numeric TI for optimal myocardial nulling in a single short breath-hold, reducing operator error and improving robustness for LGE imaging.

